# The role of calcium-dependent protein kinase in hydrogen peroxide, nitric oxide and ABA-dependent cold acclimation

**DOI:** 10.1093/jxb/ery212

**Published:** 2018-06-01

**Authors:** Xiangzhang Lv, Huizi Li, Xiaoxiao Chen, Xun Xiang, Zhixin Guo, Jingquan Yu, Yanhong Zhou

**Affiliations:** 1 Department of Horticulture, Zijingang Campus, Zhejiang University, Hangzhou, P.R. China; 2 Zhejiang Provincial Key Laboratory of Horticultural Plant Integrative Biology, Hangzhou, P.R. China

**Keywords:** Abscisic acid, calcium-dependent protein kinase, cold acclimation, hydrogen peroxide, mitogen-activated protein kinase, nitric oxide, *Solanum lycopersicum*

## Abstract

Cold acclimation-induced cold tolerance is associated with the generation of reactive oxygen species (ROS), nitric oxide (NO), and mitogen-activated protein kinases (MPKs) in plants. Here, we hypothesized that calcium-dependent protein kinases (CPKs) induce a crosstalk among ROS, NO, and MPKs, leading to the activation of abscisic acid (ABA) signaling in plant adaptation to cold stress. Results showed that cold acclimation significantly increased the transcript levels of *CPK27* along with the biosynthesis of ABA in tomato (*Solanum lycopersicum*). Silencing of *CPK27* compromised acclimation-induced cold tolerance, generation of hydrogen peroxide (H_2_O_2_) in the apoplast, NO and ABA accumulation, and the activation of MPK1/2. Crosstalk among H_2_O_2_, NO, and MPK1/2 contributes to the homeostasis of H_2_O_2_ and NO, activation of MPK1/2, and cold tolerance. ABA is also critical for CPK27-induced cold tolerance, generation of H_2_O_2_ and NO, and the activation of MPK1/2. These results strongly suggest that CPK27 may function as a positive regulator of ABA generation by activating the production of ROS and NO as well as MPK1/2 in cold adaptation.

## Teaser

Silencing the calcium-dependent protein kinase *CPK27* in tomato decreases cold-induced ABA, H_2_O_2_ and NO production, as well as decreasing activation of mitogen-activated protein kinase, compromising cold acclimation.

## Introduction

Plants undergo continuous exposure to various biotic and abiotic stresses in their growth environment. Cold stress adversely affects the growth and development of plants and significantly constrains their spatial distribution and agricultural productivity. To survive under stressful conditions, plants have evolved intricate mechanisms to perceive external signals, allowing optimal responses to environmental conditions. For example, many plants show decreased sensitivity to cold stress when they have undergone prior exposure to suboptimal low temperatures, a phenomenon called cold acclimation. Cold acclimation involves the remodeling of cell and tissue structures and the reprogramming of metabolism and gene expression (Thomashow, 1998; Chinnusamy *et al.*, 2007). The molecular basis of this acquired cold tolerance is not completely understood.

Generally, a stress signal is first perceived by a membrane receptor, followed by the up-regulation of the cytoplasmic Ca^2+^ ion level (McAinsh and Hetherington, 1998). This change in cytoplasmic Ca^2+^ is sensed by calcium sensors such as calcium-dependent protein kinases (CPKs), which interact with downstream signaling components including hormones such as abscisic acid (ABA), reactive oxygen species (ROS), and mitogen-activated protein kinases (MPKs), leading to an increased tolerance to various stresses (Boudsocq and Sheen, 2013). CPKs play an important role in signal transduction, and the functions of several CPKs in Arabidopsis and rice have recently been identified (Almadanim *et al.*, 2017; Jin *et al.*, 2017). Activated CPKs participate in different signal transduction pathways through the phosphorylation of specific substrates (Kobayashi *et al.*, 2012; Santin *et al.*, 2017). Recently, several CPK substrates have been identified. For instance, MPK5 is directly phosphorylated by CPK18 to regulate stress responses and disease resistance in rice, while StCDPK5 and AtCPK1/2/4/5/11 can phosphorylate and thereby activate NADPH oxidase to promote the production of ROS in response to abiotic and biotic stimuli (Kobayashi *et al.*, 2007; Dubiella *et al.*, 2013; Gao *et al.*, 2013; Xie *et al.*, 2014).

The tight regulation of steady-state levels of ROS is important for many cellular processes in plants (Fujita *et al.*, 2006). While excess ROS accumulation induces oxidative stress in cells, ROS also function as signaling molecules (Fujita *et al.*, 2006). Recent studies suggest that the NADPH-dependent respiratory burst oxidase homolog (RBOH) genes are involved in cold acclimation-induced cold tolerance in several plants. Silencing of *RBOH1* in tomato increased the sensitivity of plants to cold and attenuated the protective role of cold acclimation (Zhou *et al.*, 2012). Similar to ROS, nitric oxide (NO) also participates in the regulation of cold response in several plant species (Zhao *et al.*, 2009; Diao *et al.*, 2017). In particular, nitrate reductase (NR)-dependent NO participates in the regulation of the cold acclimation process (Zhao *et al.*, 2009; Lv *et al.*, 2017). In a number of cases, generation of NO and H_2_O_2_ occurs in parallel or in rapid succession, and these molecules can act synergistically or independently (Bright *et al.*, 2006; Asai *et al.*, 2008; Shi *et al.*, 2015; Deng *et al.*, 2016). In addition, increased generation of ROS/NO is frequently accompanied by the activation of MPKs. Silencing of *MPK1* and *MPK2* compromised cold acclimation-induced chilling tolerance and the activation of several antioxidant enzymes in tomato (Lv *et al.*, 2017). However, the role of CPKs in regulating the generation of ROS/NO and the activation of MPKs is unclear.

As a phytohormone, ABA is extensively involved in the responses to abiotic stresses such as drought, low temperature, and osmotic stress. In response to cold stress, plants usually accumulate an increased amount of ABA. Many stress-inducible genes are regulated by the endogenous ABA that accumulates during stress (Shinozaki *et al.*, 2003). Plants deficient or impaired in ABA biosynthesis or signaling components show a reduced response to cold acclimation (Gilmour and Thomashow, 1991; Mantyla *et al.*, 1995). ABA can also act as a secondary signal to induce changes in Ca^2+^ levels that eventually impact cold signaling. ABA-enhanced stress tolerance is associated with the induction of diverse signaling molecules, such as Ca^2+^, calmodulin (CaM) (Hu *et al.*, 2007), H_2_O_2_ (Jiang and Zhang, 2002), NO (Neill *et al.*, 2008), MPK (Zhang *et al.*, 2006; Xing *et al.*, 2008), and CPK (Ding *et al.*, 2013; Zhu *et al.*, 2007; Zou *et al.*, 2015), suggesting an intensive crosstalk among ROS, NO, and MPKs. However, how plants transduce calcium signaling to affect ROS, NO, and MPK signaling remains largely unclear, as does the relation of CPKs to ABA in cold acclimation.

Here, we hypothesized that CPKs induce a crosstalk among ROS, NO, and MPKs, leading to the activation of ABA signaling in plant adaptation to cold stress. To this end, we identified the critical CPKs in the cold response and examined their relation to the induction of H_2_O_2_, NO, and ABA signaling, and the activation of MPK1/2. Results showed that CPK27-mediated crosstalk between H_2_O_2_, NO, and MPK1/2 contributed to cold acclimation-induced ABA biosynthesis and cold tolerance in tomato plants. We also found that crosstalk between CPK27 and ABA was essential for the tight control of the interactions of H_2_O_2_ with NO and MPK1/2.

## Materials and methods

### Plant material and growth conditions

In the current study, tomato (*Solanum lycopersicum* L.) cultivar Condine Red was used for the majority of experiments. Seedlings were grown in a mixture of vermiculite and peat at the ratio of 3:1 (v/v), receiving Hoagland’s nutrient solution daily. The growth conditions were as follows: 12 h photoperiod, temperature of 25/20 °C (day/night), and photosynthetic photon flux density (PPFD) of 600 µmol m^−2^ s^−1^. To examine the significance of endogenous ABA levels in cold acclimation-induced cold tolerance, seeds of wild type (WT, cv. Ailsa Craig) and an ABA biosynthesis mutant, *notabilis* (*not*), in the Ailsa Craig background were used.

Virus-induced gene silencing (VIGS) was performed on the fully expanded cotyledons of 15-day-old tomato seedlings as described previously (Lv *et al.*, 2017). The fragment for silencing *CPK27* was PCR-amplified using the gene-specific primer listed in Supplementary Table S1 at *JXB* online, and the vectors for silencing *NR* and *RBOH1* and co-silencing *MPK1/2* were constructed as described previously (Kandoth *et al.*, 2007; Zhou *et al.*, 2014*b*; Lv *et al.*, 2017). The infiltrated plants were grown at 21 °C and used in experiments at the five-leaf stage. qRT-PCR analysis was performed to evaluate gene silencing efficiency (Supplementary Fig. S1).

### Cold acclimation and cold stress treatments

All experiments were carried out in environmentally controlled growth chambers using tomato plants at the five-leaf stage. For the cold acclimation treatment, plants were exposed to an aerial temperature of 12 °C with a 12/12 h light/dark cycle under 600 µmol m^−2^ s^−1^ PPFD and 85% humidity from 07.00 h. The cold acclimation treatment lasted for 3 d. Then, acclimated and non-acclimated plants were challenged with a cold stress treatment of 4 °C with 12/12 h light/dark cycle for 5 d under 600 μmol m^−2^ s^−1^ PPFD. There were four replicates in each treatment, and each replicate consisted of 16 plants.

To determine the roles of H_2_O_2_, NO, and ABA in cold acclimation-induced cold tolerance, tomato seedlings at the five-leaf stage were sprayed with 10 mM H_2_O_2_, 500 μM sodium nitroprusside (SNP; a donor of NO) or 50 μM ABA. H_2_O_2_ and SNP were diluted with water while ABA was dissolved in a very low concentration of ethanol (0.01%, v/v) in all working solutions including control water solution. Twelve hours after the pretreatment, the plants were transferred to growth chambers for the cold acclimation treatment at 12 °C for 3 d and then exposed to 4 °C for 5 d.

### Evaluation of cold tolerance

An Imaging-PAM Chlorophyll Fluorimeter equipped with a computer-operated PAM-control unit (IMAG-MAXI; Heinz Walz, Effeltrich, Germany) was used to detect the maximum quantum yield of PSII (*F*_v_/*F*_m_), as previously described (Zhou *et al.*, 2012). The air temperature, relative humidity, CO_2_ concentration and PPFD were maintained at 25 °C, 85%, 380 μmol mol^−1^ and 1200 μmol m^−2^ s^−1^, respectively. Relative electrolyte leakage (REL, %) was determined following protocols described previously (Cao *et al.*, 2007).

### Measurements of endogenous NO level and NR activity

NO accumulation in leaves was monitored using the NO-sensitive dye 4-amino-5-methylamino-2′,7′-difluorofluorescein diacetate (DAF-2DA) as reported previously (Lv *et al.*, 2017). Leaf sections were infiltrated with 10 µM DAF-2DA prepared in 10 mM Tris–HCl (pH 7.4) and incubated in the dark at 37 °C for 30 min before observation. Fluorescence from DAF-2T, the reaction product of DAF-2DA with NO, was observed using a fluorescence stereomicroscope (Leica TCS SL; Leica Microsystems, Wetzlar, Germany) equipped with a charge-coupled device camera. The excitation was at 488 nm, and emission images at 525 nm were obtained with a constant acquisition time. The treatments were repeated 10 times. The signal intensities of green fluorescence in the images were quantified by ImageJ software (NIH, Bethesda, MD, USA). Values were corrected for background. Data were presented as the means of fluorescence intensity relative to those of control plants. In addition, NO accumulation was also determined by colorimetric assay with Griess reagent. Briefly, 0.3 g tomato leaves were homogenized using 50 mM glacial acetic acid (pH3.6) in an ice bath and centrifuged at 12000 *g* for 15 min. An aliquot of supernatant was mixed with Griess reagent (Sigma-Aldrich, USA) and kept at 25 °C for 30 min for reaction. The content of NO was calculated based on the absorbance of the reaction mixture at 540 nm. NO content was calculated by comparison with a standard curve of NaNO_2_.

NR was assayed as described by Foyer *et al.* (1998) with small modifications. Briefly, 0.3 g leaf tissue was homogenized with 1.5 ml of extraction buffer [10 mM HEPES–KOH, pH 7.5, 5 mM DTT, 1 mM Na_2_MoO_4_, 10 mM FAD, 2 mM β-mercaptoethanol, 5 mM EDTA, and 1% polyvinylpolypyrrolidone (PVP)]. After centrifuging at 4 °C for 15 min at 12000 *g*, 0.3 ml of the supernatant was used for the NR activity assay. The reaction mixture (0.7 ml) consisted of 100 mM HEPES–KOH buffer, pH 7.5, 5 mM KNO_3_, and 0.25 mM NADH (freshly prepared). The reaction was terminated after 25 min by adding an equal volume of sulfanilamide [1% (w/v) in 3 M HCl] and then *N*-(1-naphthyl)-ethylenediamine dihydrochloride [0.02% (w/v)] to the reaction mixture, and the absorbance was measured using a spectrophotometer at 540 nm. The protein content was determined following the Coomassie blue staining method as previously described (Bradford, 1976). The NR activity was expressed as nmol of NO_2_^−^ produced per minute and per milligram of protein.

### Measurements of endogenous H_2_O_2_ levels and NADPH oxidase activity

H_2_O_2_ was extracted from leaf tissue according to Doulis *et al.* (1997) and measured as described in our previous study (Xia *et al.*, 2011). For the determination, 0.3 g FW leaf material was homogenized with 3 ml of 0.2 M HClO_4_ on ice. After centrifuging at 6000 *g* for 5 min at 4 °C, the supernatant was adjusted to pH 6.5 with 4 M KOH, and centrifuged at 12 000 *g* for 5 min at 4 °C. After filtering through an AG1 × 8 prepacked column (Bio-Rad, Hercules, CA, USA), 800 µl of extracts was mixed with 400 µl reaction buffer containing 4 mM 2,2′-azino-di (3-ethylbenzthiazoline-6-sulfonic acid) and 100 mM potassium acetate at pH 4.4, 400 µl deionized water and 0.25 U of horseradish peroxidase (HRP). H_2_O_2_ content was measured at optical density at 412 nm.

H_2_O_2_ was visualized at the subcellular level using CeCl_3_ for localization (Zhou *et al.*, 2012). Tissue pieces (1–2 mm^2^) were cut from the leaves and incubated in freshly prepared 5 mM CeCl_3_ in 50 mM MOPS at pH 7.2 for 1 h. The leaf sections were then fixed in 1.25% (v/v) glutaraldehyde and 1.25% (v/v) paraformaldehyde in 50 mM sodium cacodylate buffer (pH 7.2) for 1 h. After fixation, tissues were washed twice for 10 min in the same buffer and postfixed for 45 min in 1% (v/v) osmium tetroxide and then dehydrated in a graded ethanol series (30–100%; v/v) and embedded in Eponaraldite (Agar Aids). After 12 h in pure resin, followed by a change of fresh resin for 4 h, the samples were polymerized at 60 ℃ for 48 h. Blocks were sectioned (70–90 nm) on a Reichert-Ultracut E microtome and mounted on uncoated copper grids (300 mesh). The CeCl_3_ deposits formed in the presence of H_2_O_2_ were examined using a transmission electron microscope (H7650, Hitachi, Tokyo, Japan) at an accelerating voltage of 75 kV (Bestwick *et al.*, 1997).

For the determination of NADPH oxidase activity, leaf plasma membranes were isolated using a two-phase aqueous polymer partition system (Xia *et al.*, 2009). Briefly, leaves (5 g) were homogenized in 20 ml extraction buffer [50 mM Tris–HCl, pH 7.5, 0.25 M sucrose, 1 mM ascorbic acid (AsA), 1 mM EDTA, 0.6% PVP, and 1 mM phenylmethylsulfonyl fluoride (PMSF)]. The homogenate was filtered through four layers of cheesecloth, and the resulting filtrate was centrifuged at 10000 *g* for 15 min. Microsomal membranes were pelleted from the supernatant by centrifugation at 100000 *g* for 30 min. The pellet was suspended in 0.33 M sucrose, 3 mM KCl, and 5 mM potassium phosphate, pH 7.8. The plasma membrane fraction was isolated by adding an aqueous two-phase polymer system to give a final composition of 6.2% (w/w) dextran T500, 6.2% (w/w) polyethylene glycol 3350, 0.33 M sucrose, 3 mM KCl, and 5 mM potassium phosphate, pH 7.8. Three successive rounds of partitioning yielded the final upper phase. The upper phase was diluted 5-fold in Tris–HCl dilution buffer (10 mM, pH 7.4) containing 0.25 M sucrose, 1 mM EDTA, 1 mM DTT, 1 mM AsA, and 1 mM PMSF. The fractions were centrifuged at 120000 *g* for 1 h. The pellets were then resuspended in Tris–HCl dilution buffer and used immediately for further analysis. The protein content was determined following the Coomassie blue staining method as previously described (Bradford, 1976). The NADPH-dependent O_2_^−^-generating activity in isolated plasma membrane vesicles was determined as described previously (Xia *et al.*, 2009). The rates of O_2_^−^ generation were calculated using an extinction coefficient of 21.6 mM^−1^ cm^−1^.

### Total RNA extraction and qRT-PCR analysis

Total RNA was extracted using an RNAsimple Total RNA Kit (Tiangen, Beijing, China) following the manufacturer’s protocols. After removing residual DNA with a DNase Mini Kit (Qiagen, Hilden, Germany), total RNA was reverse transcribed using a ReverTra Ace qPCR RT Kit (Toyobo, Osaka, Japan), following the manufacturer’s instructions. The gene-specific primer pairs used to amplify the 29 *CPK* genes were taken from a previously published study (Hu *et al.*, 2016), and additional primers are shown in Supplementary Table S2. qRT-PCR was performed using a Roche LightCycler 480 real-time PCR machine (Roche, Basel, Switzerland). Relative transcript levels were calculated according to the method of Livak and Schmittgen (2001). The tomato *ACTIN2* gene was used as an internal reference.

### Total protein extraction and western blot analysis

Total protein extracts were obtained by homogenizing frozen powdered leaves in an extraction buffer (100 mM HEPES, pH 7.5, 5 mM EDTA, 5 mM EGTA, 10 mM DTT, 10 mM Na_3_VO_4_, 10 mM NaF, 50 mM β-glycerophosphate, 1 mM PMSF, 5 μg ml^−1^ antipain, 5 μg ml^−1^ aprotinin, 5 μg ml^−1^ leupeptin, 10% glycerol, and 7.5% PVP). The extracts were centrifuged at 12000 *g* for 20 min before quantifying total protein content. For immunoblots, protein extracts were resolved on a 12.5% SDS-PAGE gel and later transferred to polyvinylidene fluoride membranes (Bio-Rad). The activated state of MPK1/2 was detected using an anti-p42/44 MPK primary antibody (1:1000, Cell Signaling Technology, Boston, MA, USA) followed by anti-rabbit HRP-conjugated secondary antibodies (Cell Signaling Technology, Boston, MA, USA) (Zhou *et al.*, 2014*b*). Accumulation of MPK1/2 was quantified using Quantity One software (Bio-Rad). To obtain a loading control, membranes were stained with Ponceau S solution.

### Measurement of ABA levels

ABA extraction and analysis from tomato leaves were performed using previously described procedures (Wang *et al.*, 2016). Briefly, 0.1 g of frozen leaf material was spiked with D6-ABA (OlChemIm Ltd, Czechoslovakia) as an internal standard to a final concentration of 100 ng ml^−1^ before extraction with 1 ml of ethyl acetate. Following shaking for 12 h in the dark at 4 °C and centrifuging at 18000 *g* for 10 min at 4 °C, the supernatant was collected and evaporated to dryness under N_2_ gas. The residue was then resuspended in 0.5 ml of 70% methanol (v/v) and centrifuged at 18000 *g* for 2 min at 4 °C, and the supernatant was analysed by high-performance liquid chromatography (HPLC)–mass spectrometry (MS) on an Agilent 1290 Infinity HPLC system (including a vacuum degasser, a binary pump, a column oven, and an autosampler) coupled to an Agilent 6460 Triple Quadrupole LC/MS system (Agilent Technologies, Heilbronn, Germany). The levels of ABA in tomato leaves were expressed as nanograms per gram fresh weight leaf material.

### Statistical analysis

The experimental design was a completely randomized block with four replicates. Data were statistically analysed by one-way ANOVA and the means were compared using Tukey’s multiple comparison test at the *P*<0.05 level.

## Results

### CPK27 is critical for cold acclimation-induced cold tolerance and ABA biosynthesis

**Fig. 1.**
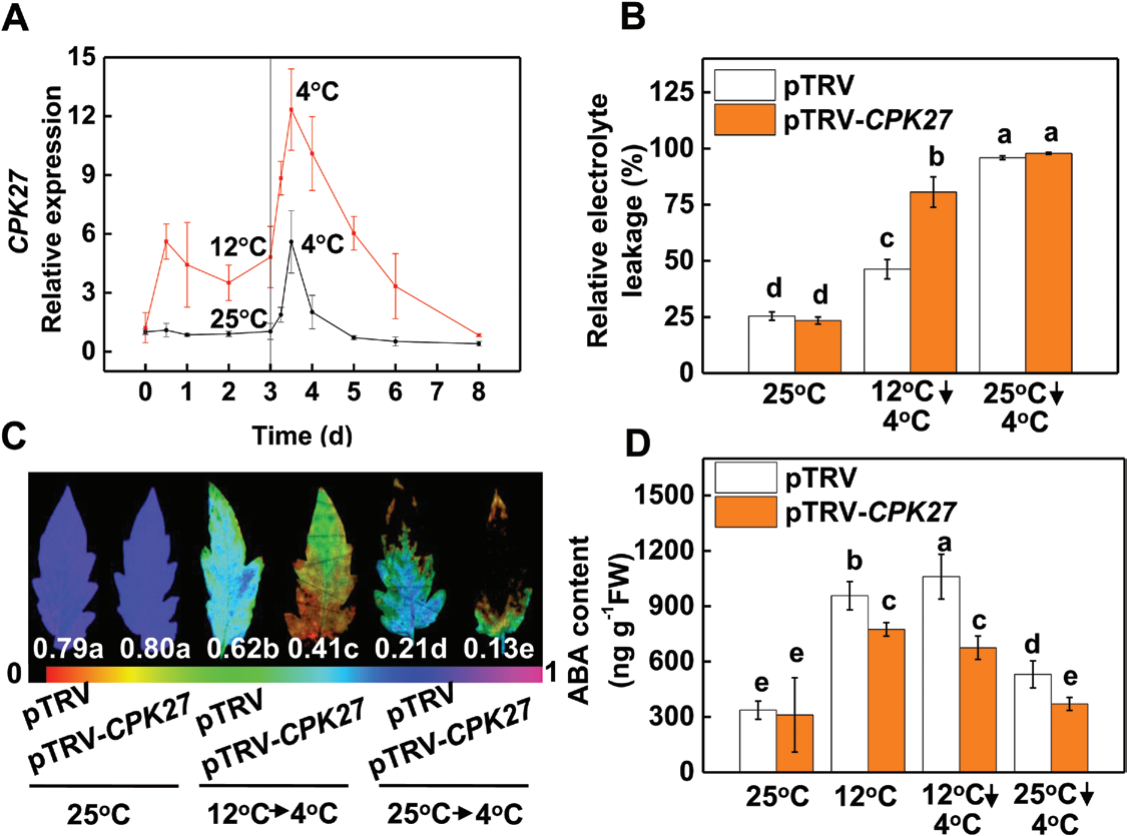
CPK27 plays a vital role in cold acclimation-induced cold tolerance. (A) Time course of changes in transcript levels of *CPK27* in response to cold stress with or without cold acclimation. (B) Relative electrolyte leakage (REL). (C) Maximum photochemical efficiency of photosystem II (*F*_v_/*F*_m_). (D) ABA content in the leaves of control (25 °C) and chilled (4 °C) tomato plants with or without cold acclimation. At the five-leaf stage, tomato plants were either cold acclimated (12 °C for 3 d) or kept at normal temperature (25 °C) before the imposition of cold stress (4 °C for 5 d). *CPK27* gene expression was analysed at different time points as indicated in (A). REL and *F*_v_/*F*_m_ values were determined at 5 d, while ABA content was assayed at 12 h after commencement of the cold stress treatment. The false color code depicted at the bottom of each image ranges from 0 (black) to 1.0 (purple), representing the level of leaf damage. Data are the means (±SD) of four biological replicates, except for *F*_v_/*F*_m_, which represents the mean of 15 leaves from independent plants. Different letters indicate significant differences (*P*<0.05) according to Tukey’s test.

In our previous study, we identified 29 *CPK* genes in tomato (Hu *et al.*, 2016). To examine the potential roles of these CPKs in cold tolerance, we examined the changes in the transcript levels of these 29 *CPK*s in response to cold acclimation in the leaves by qRT-PCR. After cold acclimation at 12 °C for 3 d, followed by a cold stress treatment at 4 °C for 12 h, 14 out of the 29 CPKs genes showed increased transcript accumulation in the leaves, ranging from 2- to 12-fold (see Supplementary Fig. S2A). Among them, the transcript accumulation of *CPK27* (accession number: Solyc11g065660.1.1) was most highly induced, with an increase of 12-fold relative to that of plants grown at 25 °C. A time course further revealed that the transcript level of *CPK27* was increased within 0.5 d after cold exposure and remained high throughout the cold acclimation process (Fig. 1A). Moreover, a further increase in the *CPK27* transcript level was found in plants when they were subsequently exposed to cold at 4 °C for 12 h. However, after exposure to 4 °C for 5 d, the *CPK27* transcript level returned to values close to those of the controls at day 0. In comparison, the *CPK27* transcript level in non-acclimated plants was up-regulated around 4-fold at 12 h after being exposed to 4 °C and then decreased to values lower than those of the controls at 5 d after the onset of cold stress at 4 °C (Fig. 1A).

To determine the role of *CPK27* in the cold response, we silenced *CPK27* (pTRV-*CPK27*) by the VIGS approach, which reduced its transcript level by ca. 70% (see Supplementary Fig. S1C). In the *CPK27*-silenced plants, the expression of *CPK22* and *CPK26*, two close homologs of *CPK27*, was not significantly changed. Under optimal growth conditions, the empty vector plants (pTRV) and the pTRV-*CPK27* plants showed similar plant growth, maximum quantum yields of photosystem II (PSII) (*F*_v_/*F*_m_) and relative electrolyte leakage (REL) (Fig. 1B, C; Supplementary Fig. S2B). Notably, cold acclimation increased cold tolerance, as evidenced by fewer wilting symptoms, increased *F*_v_/*F*_m_, and decreased REL values relative to those of non-acclimated plants. However, no significant difference in REL was found between non-acclimated pTRV plants and non-acclimated pTRV-*CPK27* plants while *F*_v_/*F*_m_ was higher in pTRV plants than that in pTRV-*CPK27* plants (Fig. 1B, C). Importantly, *CPK27* silencing attenuated the effects of cold acclimation on cold tolerance, as evidenced by a lower *F*_v_/*F*_m_ and an increased REL relative to those of pTRV plants after the cold stress at 4 °C. In addition, ABA accumulation was increased during the cold acclimation process and subsequent cold treatment in pTRV plants, and this increase in ABA accumulation was partly attenuated in pTRV-*CPK27* plants (Fig. 1D). However, sudden cold treatment induced ABA accumulation to a lesser extent compared with cold acclimation in both pTRV plants and pTRV-*CPK27* plants.

### CPK27 triggers the generation of H_2_O_2_ and NO and the activation of MPK1/2

**Fig. 2.**
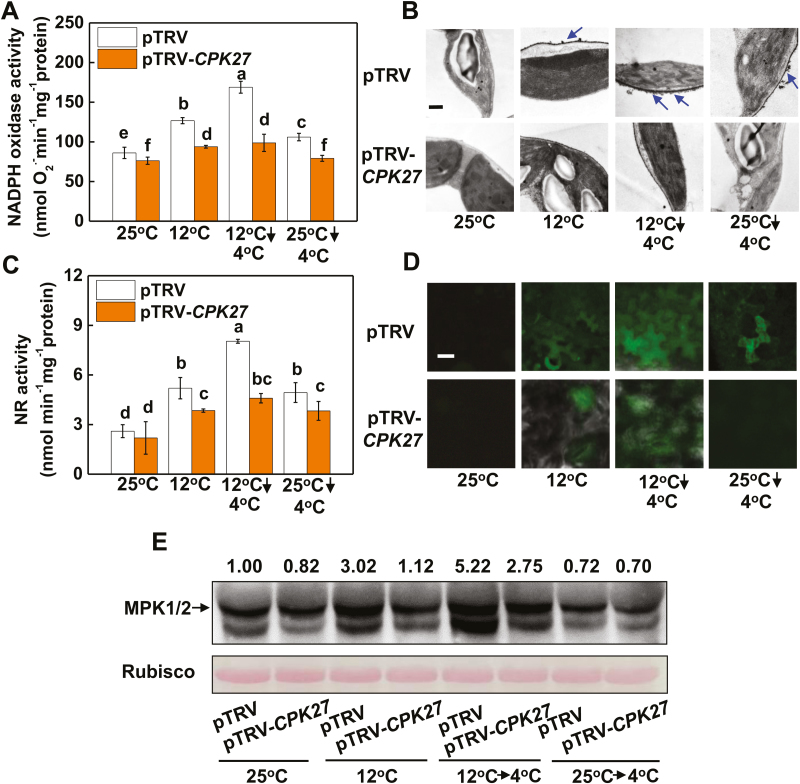
Silencing of *CPK27* attenuates NADPH-oxidase-induced H_2_O_2_ production, nitrate reductase (NR)-induced nitric oxide (NO) production and MPK1/2 activation level in tomato plants. NADPH oxidase activity (A), H_2_O_2_ accumulation in the apoplast (B), NR activity (C), NO accumulation (D), and MPK1/2 activation level (E) in the leaves of control (25 °C) and chilled (4 °C) tomato plants with or without cold acclimation. H_2_O_2_ accumulation in the apoplast was assayed by observing CeCl_3_ precipitates located on the membrane using a transmission electron microscope. Blue arrows indicate apoplastic H_2_O_2_ accumulation; scale bar: 1 μm. NO accumulation in leaves was visualized using an NO-specific fluorescent probe, 4-amino-5-methylamino-2′,7′-difluorofluorescein diacetate (DAF-2DA), and fluorescence was photographed with a laser scanning confocal microscope (LSCM-500, Zeiss, Germany). Representative images selected from 10 replicates of each treatment are shown in (D); scale bar: 50 μm. The numbers above the images in (E) indicate the relative intensity of MPK1/2 (the upper lane). All parameters in this figure were assayed at 12 h after commencement of the cold stress treatment. Data are the means (±SD) of four biological replicates. Different letters indicate significant differences (*P*<0.05) according to Tukey’s test.

NO, H_2_O_2_, and MPKs are all involved in cold acclimation-induced cold tolerance (Zhao *et al.*, 2009; Cantrel *et al.*, 2011; Zhou *et al.*, 2012). We thus examined whether cold acclimation-induced accumulation of NO and H_2_O_2_ and the activation of MPK were *CPK27* dependent. At 25 °C, no significant differences in the transcript levels of *RBOH1*, *NR*, *MPK1*, and *MPK2* (see Supplementary Fig. S3A–D), the activities of NADPH oxidase and NR (Fig. 2A, C), or the accumulation levels of H_2_O_2_ and NO (Fig. 2B, D; Supplementary Fig. S3E–G) were found between pTRV plants and pTRV-*CPK27* plants. Both cold acclimation and cold stress increased the transcript levels of *RBOH1*, *NR*, *MPK1*, and *MPK2*, the activities of NADPH oxidase and NR, and content of H_2_O_2_ and NO in pTRV plants (Fig. 2A–D; Supplementary Fig. S3). Subsequent exposure to cold stress further increased the transcript levels of *RBOH1*, *NR*, *MPK1*, and *MPK2*, the activities of NADPH oxidase and NR, and the accumulation levels of H_2_O_2_ and NO in acclimated plants (Fig. 2A–D; Supplementary Fig. S3). However, silencing *CPK27* significantly attenuated the cold acclimation-induced transcript levels of *RBOH1*, *NR*, *MPK1*, and *MPK2*, the activities of NADPH oxidase and NR, and the accumulation levels of H_2_O_2_ and NO (Fig. 2A–D; Supplementary Fig. S3). MPK1/2 activation (the upper lane) was induced by cold acclimation but not by sudden cold at 4 °C in pTRV and pTRV-*CPK27* plants (Fig. 2E). However, cold acclimation-induced MPK1/2 activation was attenuated in *CPK27*-silenced plants. In addition, we found that the application of H_2_O_2_ or SNP increased cold acclimation-induced cold tolerance not only in pTRV plants but also in pTRV-*CPK27* plants (Supplementary Fig. S4). All these results suggest that *CPK27* is involved in the cold acclimation-induced accumulation of H_2_O_2_ and NO and MPK1/2 activation and *CPK27* appears to act upstream of H_2_O_2_ and NO in cold adaptation.

### Relation of NO, H_2_O_2_, and MPK1/2 in cold acclimation

**Fig. 3.**
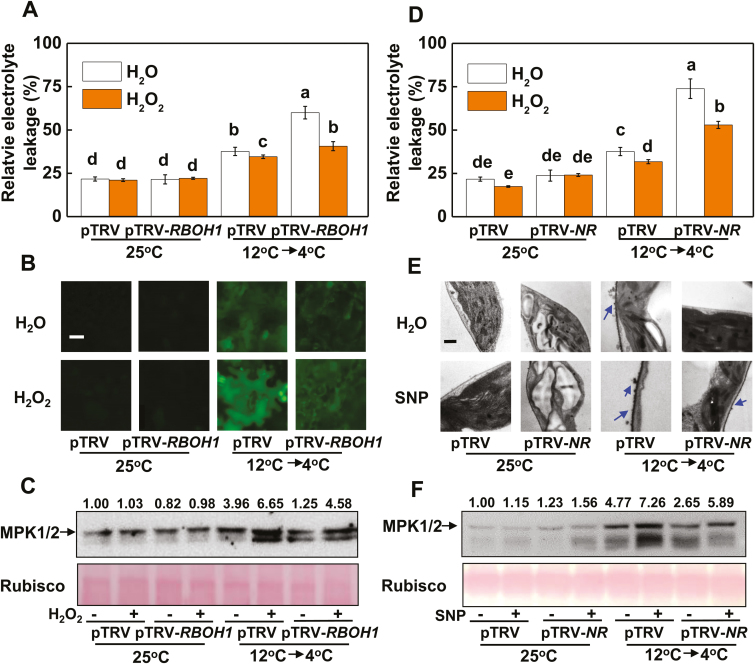
NADPH-oxidase-dependent H_2_O_2_ and nitrate reductase (NR)-dependent NO are essential for cold acclimation-induced MPK1/2 activation and cold tolerance. (A, D) REL in *RBOH1*- (A) and *NR*- (D) silenced plants. (B, E) NO accumulation in *RBOH1*-silenced plants (B) and H_2_O_2_ accumulation in the apoplast in *NR*-silenced plants (D). (C, F) MPK1/2 activation level in *RBOH1*- (C) and *NR*- (F) silenced plants. H_2_O_2_ at 10 mM and SNP at 500 μM were applied 12 h before the cold acclimation treatment. H_2_O_2_ and NO accumulation in leaves were visualized using the same protocols as shown in Fig. 2. Scale bars: 50 μm (B) and 1 μm (E). The numbers above the images in (C, F) indicate the relative intensity of MPK1/2 (the upper lane). REL was determined at 5 d, while the other parameters were assayed at 12 h after commencement of the cold stress treatment. Data are the means (±SD) of four biological replicates. Different letters indicate significant differences (*P*<0.05) according to Tukey’s test.

We next investigated how NO, H_2_O_2_, and MPK1/2 were involved in the acclimation-induced tolerance to cold. Plants with *RBOH1* silencing (pTRV-*RBOH1*) showed increased sensitivity to cold stress despite the cold acclimation treatment as evidenced by increased REL and decreased *F*_v_/*F*_m_ (Fig. 3A; Supplementary Fig. S5A). pTRV-*RBOH1* plants showed decreased accumulation of NO and reduced activation of MPK1/2 relative to those in pTRV plants after the cold acclimation (Fig. 3B, C; Supplementary Fig. S5B, C). However, foliar application of exogenous H_2_O_2_ enhanced cold tolerance as indicated by the lower REL, higher *F*_v_/*F*_m_, and increased accumulation of NO, with the effects being more significant in pTRV-*RBOH1* plants (Fig. 3A, B; Supplementary Fig. S5A–C). Exogenous H_2_O_2_ increased the activation of MPK1/2 in both pTRV plants and pTRV-*RBOH1* plants (Fig. 3C). On the other hand, plants with *NR* silencing (pTRV-*NR*) showed increased REL and decreased *F*_v_/*F*_m_ (Fig. 3D; Supplementary Fig. S5D), which was followed by decreased accumulation of H_2_O_2_ and reduced activation of MPK1/2 relative to pTRV after the cold acclimation (Fig. 3E, F; Supplementary Fig. S5E). Application of SNP enhanced cold tolerance with lower REL, higher *F*_v_/*F*_m_, and increased accumulation of H_2_O_2_ in the apoplast, with the effects being more significant in pTRV-*NR* plants (Fig. 3E; Supplementary Fig. S5E). In addition, SNP application further increased the activation of MPK1/2 in both pTRV plants and pTRV-*NR* plants (Fig. 3F). These results not only demonstrated the role of *RBOH1* and *NR* in the MPK1/2 activation and cold tolerance, but also indicated the interdependency of the production of H_2_O_2_ and that of NO during the cold acclimation.

**Fig. 4.**
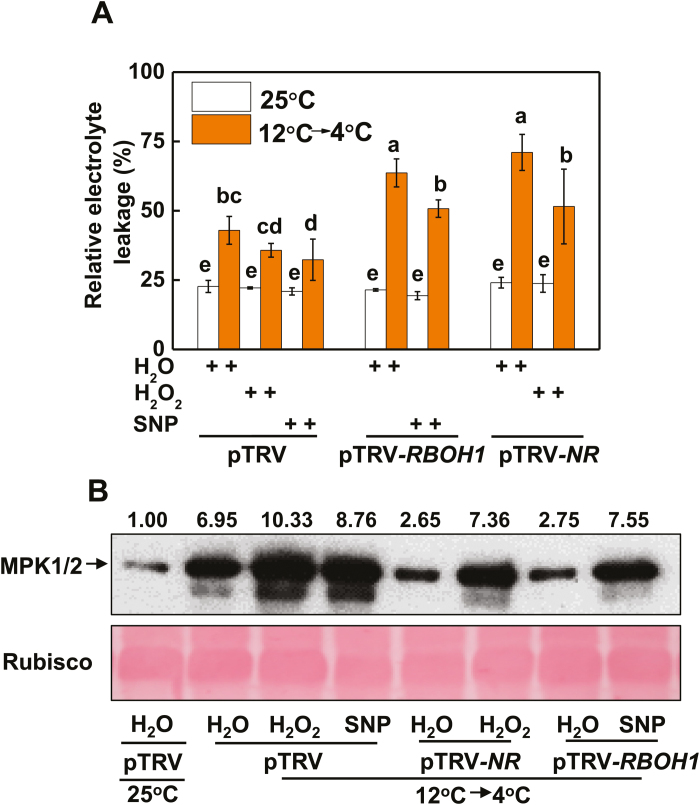
Crosstalk between H_2_O_2_ and NO and its role in the regulation of MPK1/2 activation in tomato plants. (A, B) REL (A) and MPK1/2 activation (B) in *RBOH1*- or *NR*-silenced plants sprayed with 500 μM SNP or 10 mM H_2_O_2_, respectively. pTRV plants sprayed with water at 25 °C are shown as control. The numbers above the images in (B) indicate the relative intensity of MPK1/2 (the upper lane). REL and MPK1/2 activation were assayed at 5 d and 12 h, respectively, after commencement of the cold stress treatment. Data are the means (±SD) of four biological replicates. Different letters indicate significant differences (*P*<0.05) according to Tukey’s test.

To examine the respective contribution of H_2_O_2_ and NO in acclimation-induced cold tolerance and the activation of MPK1/2, we applied either H_2_O_2_ or SNP to pTRV-*NR* and pTRV-*RBOH1* plants before they were exposed to the cold acclimation and cold stress treatments. We found that the application of H_2_O_2_ induced cold tolerance not only in pTRV plants but also in pTRV-*NR* plants as evidenced by the decreased REL (Fig. 4A). In comparison, the application of SNP decreased REL in both pTRV and pTRV-*RBOH1* plants. In agreement with these results, the application of H_2_O_2_ induced the activation of MPK1/2 in both pTRV and pTRV-*NR* plants, while the application of SNP induced the activation of MPK1/2 in both pTRV and pTRV-*RBOH1* plants (Fig. 4B). These results demonstrated that *RBOH1*-dependent H_2_O_2_ production and *NR*-dependent NO production both play a critical role in cold acclimation-induced MPK1/2 activation and cold tolerance in tomato plants. Moreover, the results indicated that H_2_O_2_ and NO could independently regulate cold acclimation-induced cold tolerance and MPK1/2 activation.

**Fig. 5.**
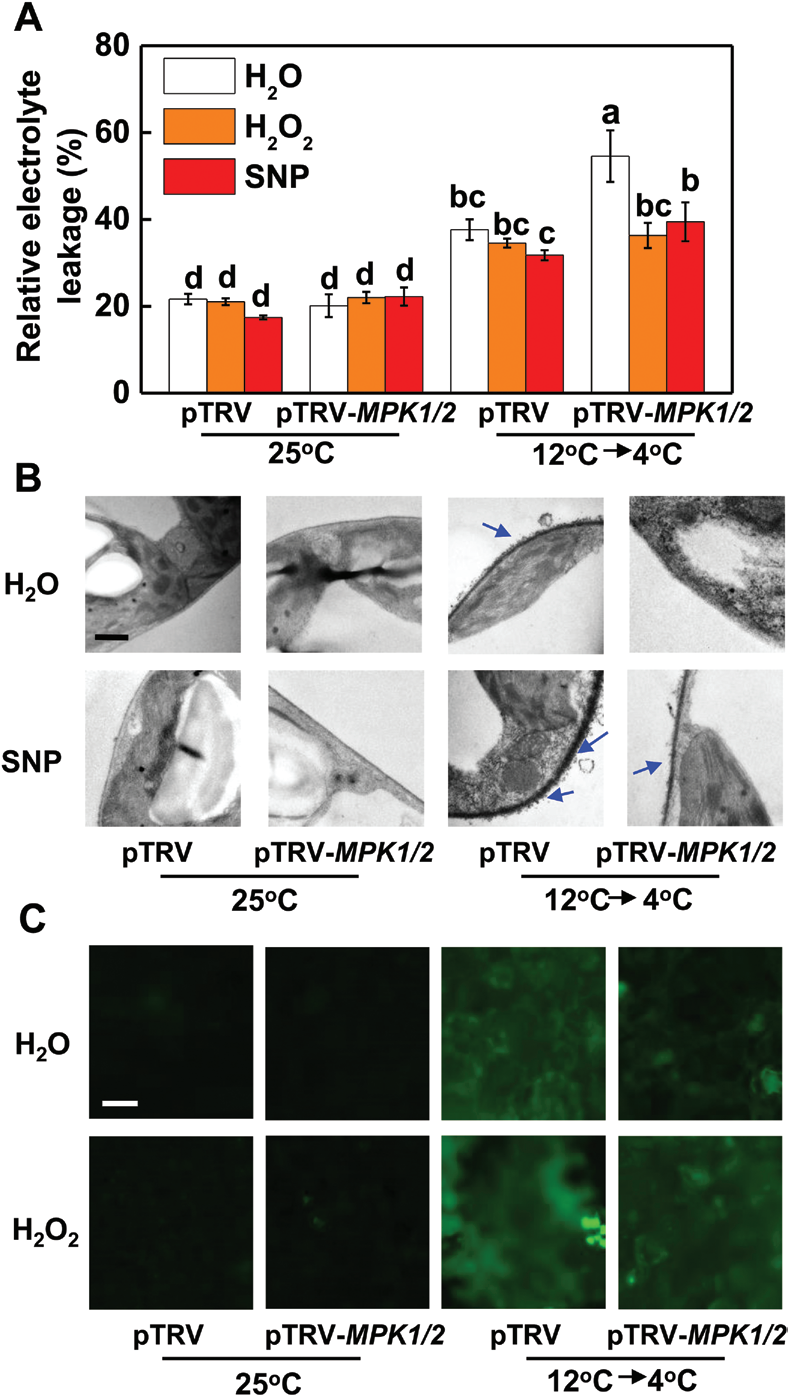
Influence of *MPK1/2* co-silencing and exogenous SNP or H_2_O_2_ on REL (A), apoplastic H_2_O_2_ (B) or NO level (C) with regard to cold acclimation-induced cold tolerance in tomato plants. SNP at 500 μM and H_2_O_2_ at 10 mM were applied 12 h before the cold acclimation treatment. Leaf samples were taken at 5 d after commencement of the cold stress treatment for the determination of REL. H_2_O_2_ and NO accumulation levels were estimated as described in Fig. 2. Scale bars: 50 μm (C) and 1 μm (B). Data are the means (±SD) of four biological replicates. Different letters indicate significant differences (*P*<0.05) according to Tukey’s test.

We then determined whether cold acclimation-activated MPK1/2 in turn affects the homeostasis of H_2_O_2_ and NO in response to cold. The results showed that *MPK1/2* silencing not only attenuated the acclimation-induced cold tolerance with higher REL and lower *F*_v_/*F*_m_, but also decreased the accumulation of H_2_O_2_ and NO (Fig. 5; Supplementary Fig. S6). Exogenous application of H_2_O_2_ and SNP restored cold tolerance, as evidenced by the decreased REL and increased *F*_v_/*F*_m_ in pTRV-*MPK1/2* plants (Fig. 5A; Supplementary Fig. S6A). Furthermore, exogenous application of SNP and H_2_O_2_ induced the accumulation of H_2_O_2_ and NO, respectively, in pTRV-*MPK1/2* plants (Fig. 5B, C; Supplementary Fig. S6B–D).

### H_2_O_2_, NO, and MPK1/2 are involved in cold acclimation-induced ABA biosynthesis

**Fig. 6.**
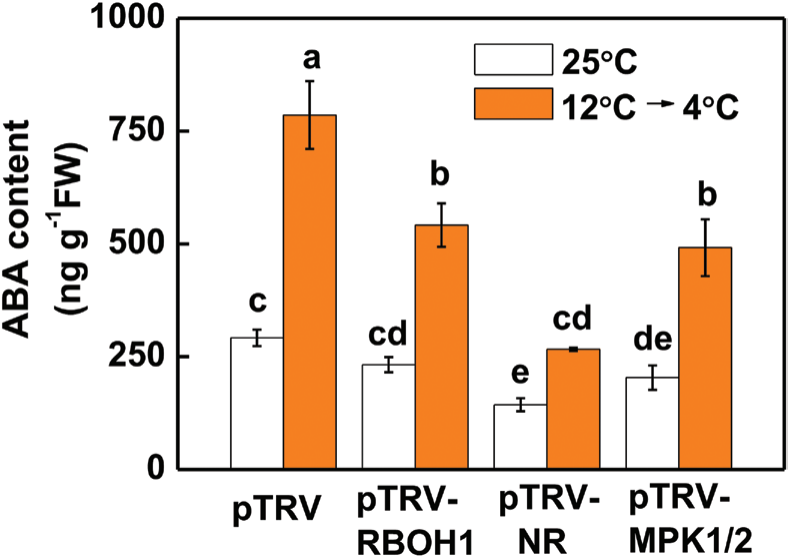
Silencing of *RBOH1*, *NR*, or *MPK1/2* attenuates cold acclimation-induced ABA accumulation. Leaf samples were taken at 12 h after commencement of the cold stress treatment for the determination of ABA content. Data are the means (±SD) of four biological replicates. Different letters indicate significant differences (*P*<0.05) according to Tukey’s test.

**Fig. 7.**
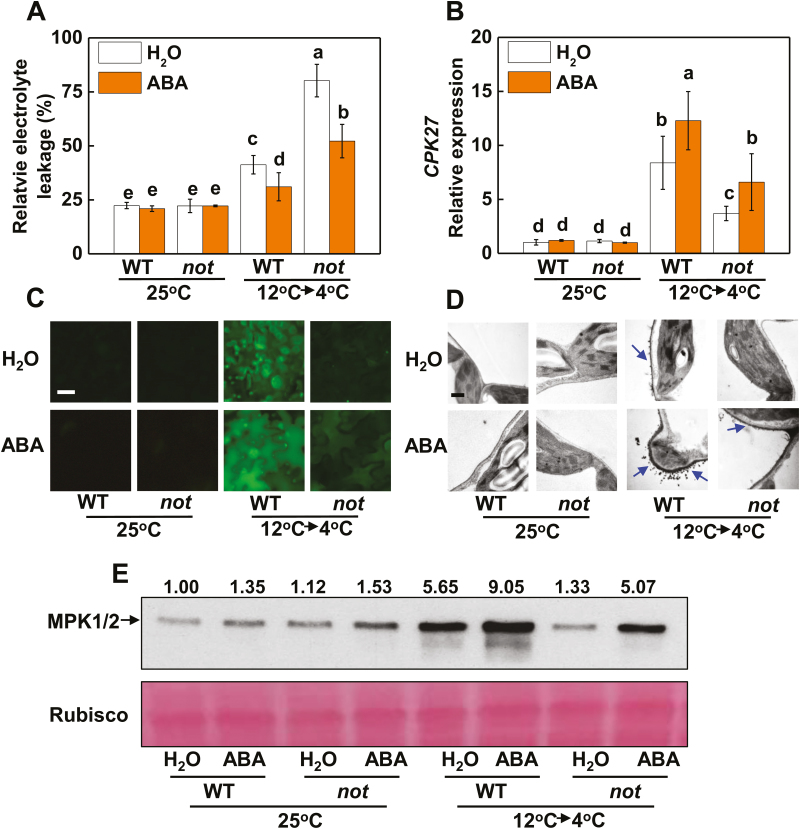
Cold acclimation-induced transcript levels of *CPK27*, accumulation of H_2_O_2_ and NO, and MPK1/2 activation are dependent on ABA levels in plants. REL (A), relative expression of *CPK27* (B), NO (C), H_2_O_2_ in the apoplast (D), and activation of MPK1/2 (E) in the wild type (WT) and the ABA biosynthetic mutant, *notabilis* (*not*), with foliar application of ABA or H_2_O. ABA at 50 μM was applied 12 h before the cold acclimation treatment. The numbers above the images in (E) indicate the relative intensity of MPK1/2 (the upper lane). Leaf samples were taken at 5 d for the determination of REL, while the other assays were performed at 12 h after commencement of the cold stress treatment. H_2_O_2_ and NO accumulation levels were estimated as described in Fig. 2. Scale bars: 50 μm (C) and 1 μm (D). Data are the means (±SD) of four biological replicates. Different letters indicate significant differences (*P*<0.05) according to Tukey’s test.

ABA is known to play important roles in plant responses to cold stress. Although ABA accumulation was induced by cold acclimation, this induction was mitigated in pTRV-*RBOH1*, pTRV-*NR*, and pTRV-*MPK1/2* plants (Fig. 6). We then investigated whether the increased accumulation of ABA was essential for the acclimation-induced cold tolerance. The tomato *notabilis* (*not*) mutant, which is deficient in ABA, showed increased cold sensitivity, as indicated by the increased REL from the leaf cells and decreased *F*_v_/*F*_m_ following exposure to cold acclimation for 3 d and cold stress conditions for 5 d (Fig. 7A; Supplementary Fig. S7A). Foliar application of exogenous ABA enhanced cold tolerance in both wild-type (WT) and *not* plants, as evidenced by the decreased REL and increased *F*_v_/*F*_m_. Cold acclimation-induced increases in *CPK27* transcript levels, accumulation of H_2_O_2_ and NO, and MPK1/2 activation were considerably attenuated in *not* plants (Fig. 7B–E; Supplementary Fig. S7B–D). Additionally, application of exogenous ABA enhanced the levels of *CPK27* transcript, H_2_O_2_ and NO, as well as MPK1/2 activation, in both WT and *not* plants.

### CPK27 is required for ABA-induced cold tolerance

**Fig. 8.**
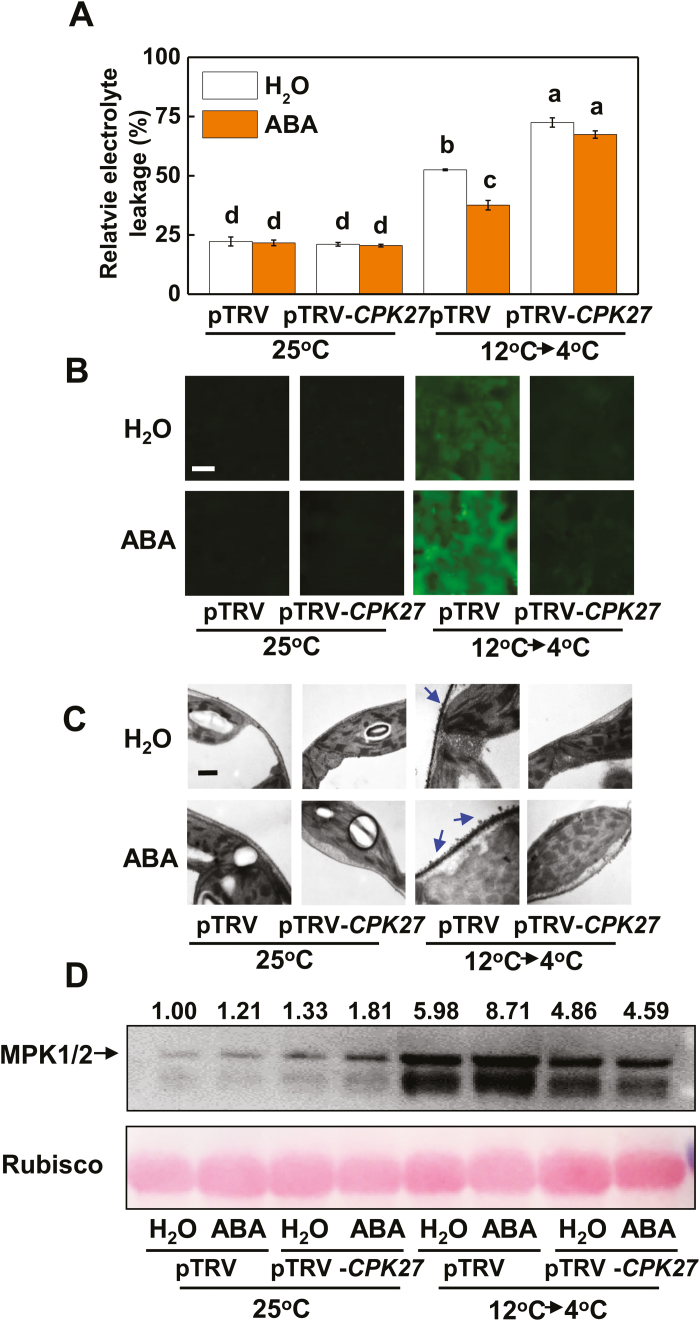
CPK27 is essential for ABA-induced cold tolerance, H_2_O_2_ and NO accumulation, and MPK1/2 activation in tomato plants. REL (A), NO (B), apoplastic H_2_O_2_ (C), and activation of MPK1/2 (D) in pTRV control plants and *CPK27*-silenced plants with foliar application of ABA or H_2_O. ABA at 50 μM was applied 12 h before the cold acclimation treatment. The numbers above the images in (D) indicate the relative intensity of MPK1/2 (the upper lane). Leaf samples were taken at 5 d for the determination of REL, while other assays were performed at 12 h after commencement of the cold stress treatment. H_2_O_2_ and NO accumulation levels were estimated as described in Fig. 2. Scale bars: 50 μm (B) and 1 μm (C). Data are the means (±SD) of four biological replicates. Different letters indicate significant differences (*P*<0.05) according to Tukey’s test.

We then examined whether cold acclimation-induced ABA accumulation regulates *CPK27*-dependent events as a feedback mechanism. Thus, we analysed the changes in cold acclimation-induced and *CPK27*-dependent accumulation of NO and H_2_O_2_, as well as MPK1/2 activation and cold tolerance, in response to exogenous ABA and cold acclimation. Application of ABA enhanced cold tolerance as indicated by the decreased REL and increased *F*_v_/*F*_m_ (Fig. 8A; Supplementary Fig. S8A), increased the accumulation of H_2_O_2_ in the apoplast and NO in the leaves, and induced MPK1/2, but these effects were greatly tempered in plants with *CPK27* silencing (Fig. 8B–D; Supplementary Fig. S8B–D). These results indicated that *CPK27* is vital for the ABA-induced production of H_2_O_2_ in the apoplast and NO in the leaves and for the induction of MPK1/2.

## Discussion

CPKs play critical roles in regulating growth, development, and stress responses in plants (Boudsocq and Sheen, 2013). There is evidence that CPKs participate in the regulation of plant response to cold stress (Martín and Busconi, 2001; Böehmer and Romeis, 2007; Komatsu *et al.*, 2007). However, the mechanisms underlying CPK-dependent cold tolerance induced by cold acclimation remain mostly elusive. Here, we present several lines of evidence supporting the role of CPK27 in the cold response. Cold acclimation-induced CPK27 contributes to early signal transduction processes and is connected to NO, H_2_O_2_, and MPK1/2 activation in ABA signaling. This study not only delineated the NO–H_2_O_2_–MPK1/2 loop as an integrated signaling mechanism for modulating ABA-dependent cold tolerance but also suggested a positive impact of ABA on CPK27-dependent cold tolerance through a feedback loop in cold acclimation.

### CPK27 is a positive regulator of cold tolerance induced by cold acclimation

Several CPKs have been shown to constitute a complicated regulation network, functioning positively and negatively in plant adaptation to cold stress. For example, OsCPK7, OsCPK13, and OsCPK17 in *Oryza sativa* (Saijo *et al.*, 2000; Komatsu *et al.*, 2007; Almadanim *et al.*, 2017), AtCPK1 in Arabidopsis (Böhmer and Romeis, 2007), PeCPK10 in *Populus euphratica* (Chen *et al.*, 2013), and VaCPK20 in *Vitis amurensis* (Dubrovina *et al.*, 2015) have been characterized as positive regulators of cold stress tolerance, while ZmCPK1 in *Zea mays* has been shown to act as a negative regulator of cold stress signaling (Weckwerth *et al.*, 2015). Here, we provide multiple lines of evidence that CPK27 is a positive regulator of the cold response in tomato plants. First, transcript levels of *CPK27* were significantly increased by cold acclimation (Fig. 1A; Supplementary Fig. S2A). Second, knockdown of *CPK27* transcripts attenuated cold acclimation-induced cold tolerance and compromised ABA-induced cold tolerance (Figs 1B, C, 8A). Third, *CPK27* silencing reduced the cold acclimation-induced accumulation of ABA, H_2_O_2_, and NO, as well as the activation of MPK1/2 (Figs 1D, 2B, D, E; Supplementary Fig. S3E–G), all of which are positive regulators of cold response in plants. These results strongly suggest that CPK27 is not only important for cold response but also involved in the regulation of multiple signaling pathways, including ABA, ROS, NO, and MPK signaling, in response to cold.

Evidence is increasing for the roles of NO, apoplastic H_2_O_2_, and MPKs in the cold response in plants (Diao *et al.*, 2017; Kim *et al.*, 2017; Si *et al.*, 2017). In agreement with our earlier results, silencing of *RBOH1* or *NR* or co-silencing of *MPK1* and *MPK2* differentially compromised cold acclimation-induced cold tolerance (Figs 3A, D, 5A). Interestingly, the transcript levels of *RBOH1*, *NR*, *MPK1*, and *MPK2* were all subject to regulation by *CPK27* (see Supplementary Fig. S3A–D). Silencing of *CPK27* attenuated cold acclimation-induced H_2_O_2_ accumulation in the apoplast, NO accumulation in the leaves, and activation of MPK1/2 (Fig. 2B, D, E; Supplementary Fig. S3E–G). These results provided convincing evidence for the role of *CPK27* in cold acclimation-triggered ROS signaling, NO signaling, and MPK signaling in the cold response. Several studies have demonstrated that CPKs can phosphorylate the N-terminal regions of plasma membrane RBOH proteins (NADPH oxidase) and participate in RBOH-mediated ROS bursts (Kobayashi *et al.*, 2012). Consistent with this, we found that the cold acclimation activation of NADPH oxidase was largely abolished in pTRV-*CPK27* plants (Fig. 2A), suggesting that the N-terminal region of NADPH oxidase is likely phosphorylated by CPK27. Evidence also exists that CPKs can activate MPK signaling by activating MPKs. For example, CPK18 in rice was identified as an upstream kinase of MAPK (MPK5) and was shown to phosphorylate and activate MPK5 (Xie *et al.*, 2014). In our present study, *CPK27* silencing greatly attenuated the cold acclimation-induced activation of MPK1/2 (Fig. 2E), suggesting a potential role of CPK27 in the activation of MPKs in tomato plants. Interestingly, cold acclimation-induced NO accumulation was also attenuated in *CPK27*-silenced plants (Fig. 2D; Supplementary Fig. S3F, G), implying the involvement of *CPK27* in the regulation of NO homeostasis by indirectly activating NR. Until now, there is no clear evidence for the direct regulation of NR by CPKs in plants *in vivo*. Further protein–protein interaction experiments will shed light on the mechanisms underlying *CPK27*-induced activation of ROS signaling, NO signaling, and MPK signaling in response to cold.

### CPK27-activated crosstalk among H_2_O_2_, NO, and MPK1/2 in cold acclimation

Several studies have demonstrated the complicated interactions between NO and H_2_O_2_, NO and MPKs, and H_2_O_2_ and MPKs in response to various stresses or stimuli (Bright *et al.*, 2006; Asai *et al.*, 2008; Takahashi *et al.*, 2011; Ye *et al.*, 2013; Zhou *et al.*, 2014*b*; Li *et al.*, 2017). Here, we provide several lines of evidence for the existence of a feedback loop among H_2_O_2_, MPKs, and NO in the cold response. First, silencing of *RBOH1* or *NR* not only abolished the accumulation of NO or H_2_O_2_, respectively, but also decreased the accumulation of activated MPK1/2 induced by cold acclimation (Fig. 3B, C, E, F). Second, foliar application of SNP to pTRV-*RBOH1* plants and foliar application of H_2_O_2_ to pTRV-*NR* plants both resulted in an increased accumulation of activated MPK1/2 (Fig. 4B). Third, silencing of *MPK1/2* decreased cold acclimation-induced accumulation of both H_2_O_2_ and NO (Fig. 5B, C). Fourth, silencing of *RBOH1*, *NR*, or *MPK1*/*2* attenuated cold acclimation-induced ABA accumulation (Fig. 6). These results allow us to argue that the crosstalk among H_2_O_2_, MPK1/2, and NO is critical to maintain the homeostasis of H_2_O_2_ and NO and the activated state of MPK1/2. However, how cold acclimation initiates this loop is unclear, as both NADPH oxidase and MPKs could be phosphorylated or activated by CPKs in plants *in vivo* (Kobayashi *et al.*, 2007; Xie *et al.*, 2014).

The H_2_O_2_–MPK1/2–NO feedback loop is likely to be important for the homeostasis of H_2_O_2_ and NO and the activation of MPK1/2. Data from the present study show that H_2_O_2_ and NO could function independently in the cold response. This finding is substantiated by the increased cold tolerance caused in pTRV-*RBOH1* by the foliar application of SNP, in pTRV-*NR* plants by the foliar application of H_2_O_2_, and in pTRV-*MPK1/2* plants by the foliar application of SNP or H_2_O_2_ (Figs 4A, 5A). While ROS, NO, and MPKs share many similarities in target genes or proteins, they can also act independently in the regulation of many physiological or metabolic processes in plants (Yoshioka *et al.*, 2011; Xu *et al.*, 2014). Studies to date support a role of ROS in the regulation of gene transcripts and protein functions by cysteine modification (Akter *et al.*, 2015). In comparison, NO and MPKs affect signaling cascades, mostly by *S*-nitrosylation and phosphorylation, respectively (Wang *et al.*, 2015; Kim *et al.*, 2017). Accordingly, the coordination of MPK1/2, H_2_O_2_, and NO likely contributes greatly to the cold acclimation-induced cold response by altering a variety of physiological processes, as substantiated by the results of RNAseq and metabolite analysis (Barrero-Gil *et al.*, 2016).

### Relationship between CPK27 and ABA in cold acclimation

Although Ca^2+^ and CPKs have been suggested as early signals in stress response, evidence for CPK activity in the induction of ABA biosynthesis or signaling is still lacking. Here, we found that CPK27 plays a critical role in ABA accumulation. We observed that cold acclimation-induced ABA accumulation was greatly attenuated in pTRV-*CPK27* plants (Fig. 1D). CPK27 is important for the acclimation-induced transcript of *RBOH1*, *NR*, and *MPK1* and *MPK2*, the generation of ROS and NO and the activation of MPK1/2 (Fig. 2; Supplementary Fig. S3). However, silencing of *RBOH1*, *NR*, or *MPK1*/*2* resulted in decreased accumulations of ABA during the cold acclimation (Fig. 6). While *RBOH1*-dependent NADPH oxidase is involved in the generation of ABA in response to heat and oxidative stresses, interaction of ROS and NO results in the induction of ABA biosynthesis (Zhao *et al.*, 2001; Xing *et al.*, 2004; Zhou *et al.*, 2014*a*). Therefore, CPK27 may function as a positive regulator of ABA generation by activating the production of ROS and NO as well as MPK1/2.

The results of the present study demonstrated that CPK27-induced generation of ROS and NO and activation of MPK1/2 were subject to regulation by ABA (Fig. 7B–E). Silencing of *CPK27* abolished the ABA-induced increase in accumulation of H_2_O_2_ and NO, and activation of MPK1/2 in response to cold acclimation (Fig. 8B–D). Many CPKs, including CPK27 in tomato, are ABA responsive (Hu *et al.*, 2016). Arabidopsis CPK4 and CPK11 have been identified as important positive regulators in CPK/calcium-mediated ABA signaling (Zhu *et al.*, 2007). ABA-induced transcriptional reprogramming via ABA-responsive ABF transcription factors is likely to be a key feature of CPK signaling. For example, AtCPK32 activates ABF4 *in vivo*, resulting in the induction of ABF4 target genes (Choi *et al.*, 2005). Taken together, these results suggest that crosstalk between ABA and CPK27 is important not only for the activation of MPK1/2, ROS signaling, and NO signaling but also for the maintenance of ABA signaling and thus for cold tolerance.

## Supplementary data

Supplementary data are available at *JXB* online.

Fig. S1. Efficiency of gene silencing by virus-induced gene silencing

Fig. S2. Effects of cold acclimation on transcripts of the tomato *CPK* family genes and plant phenotype after cold stress in tomato plants.

Fig. S3. Effects of *CPK27* silencing on the transcript levels of *RBOH1*, *NR*, *MPK1*, and *MPK2* and accumulation levels of H_2_O_2_ and NO in leaves of control (25 °C), cold-acclimated and non-acclimated tomato plants.

Fig. S4. Exogenous H_2_O_2_ or SNP partly rescues the chilling-sensitive phenotype due to *CPK27* silencing.

Fig. S5. Effects of *RBOH1* or *NR* silencing and exogenous H_2_O_2_ or SNP on cold acclimation-induced cold tolerance and NO or H_2_O_2_ accumulation in control (25 °C) and cold-acclimated tomato plants.

Fig. S6. Effects of *MPK1/2* co-silencing and exogenous SNP or H_2_O_2_ on *F*_v_/*F*_m_, accumulation of H_2_O_2_ or NO in control (25 °C) and cold-acclimated tomato plants.

Fig. S7. ABA and cold acclimation-induced changes in *F*_v_/*F*_m_ and accumulation of H_2_O_2_ and NO in the wild type and the ABA-deficient mutant *not*.

Fig. S8. ABA and cold acclimation-induced changes in *F*_v_/*F*_m_ and accumulation of H_2_O_2_ and NO in pTRV control plants and *CPK27*-silenced plants.

Table S1. PCR primer sequences used for vector construction.

Table S2. List of primer sequences used for qRT-PCR analysis.

Supplementary Tables S1-S2

Supplementary Figures S1-S8
