# Supplementary material for: The role of calcium-dependent protein kinase in hydrogen peroxide, nitric oxide and ABA-dependent cold acclimation
Source: J Exp Bot. 2018 Jun 1;69(16):4127–39. doi: 10.1093/jxb/ery212 (PMC6054180; doi:10.1093/jxb/ery212)
Supplement: Supplementary Tables S1-S2 [file ery212_suppl_supplementary_tables_s1-s2.pdf]

Table S1. PCR primer sequences used for vector construction.

Vector	primer	
pTRV2-*CPK27*	**Forward**	5′ -ggcgcgagctcGAATTGCTGTGGGACACCTG-3′
	**Reverse**	5′ -cggcgctcgagTAGCTCACAACCAAGATCATAT-3′

Table S2. List of primer sequences used for qRT-PCR analysis.

Gene	Accession number	Forward primer (5'-3')	Reverse primer (5'-3')
NR	Solyc11g013810.1.1	ATTTGGAACGTCATGGGAAT	GCCTTATCAG AATGAATTGC
RBOH1	Solyc08g081690.2.1	GTCGTGTTTGGAAGGGAACT	ATGTCCGCAAGTGTCATGTT
MPK1	Solyc12g019460.1.1	GCTGACAGATTGTTGCAGGT	TCCACCCCATAAAGATACATCA
MPK2	Solyc08g014420.2.1	TACTCGCTCGTTTGCTGTTG	TTGGAGTACAGGAAAACAATGG
ACTIN2	Solyc11g005330.1.1	TGTCCCTATTTACGAGGGTTATG	CAGTTAAATCACGACCAGCAAGA
