# Supplementary material for: The role of calcium-dependent protein kinase in hydrogen peroxide, nitric oxide and ABA-dependent cold acclimation
Source: J Exp Bot. 2018 Jun 1;69(16):4127–39. doi: 10.1093/jxb/ery212 (PMC6054180; doi:10.1093/jxb/ery212)
Supplement: Supplementary Figures S1-S8 [file ery212_suppl_supplementary_figures_s1-s8.pdf]

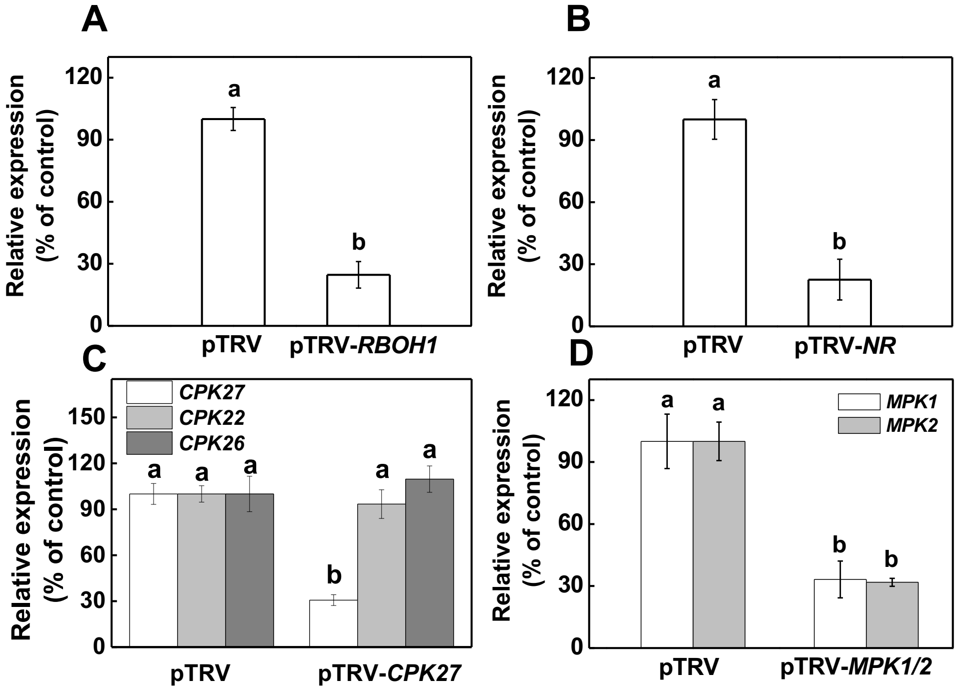

Fig. S1. Efficiency of gene silencing by virus-induced gene silencing (VIGS). (A) Efficiency of *RBOH1* silencing. (B) Efficiency of *NR* silencing. (C) Efficiency of *CPK27* silencing and its influence on expression of 2 close homologs *CPK22* and *26*. (D) Efficiency of *MPK1* and *MPK2* silencing. Data are mean±SD of four biological replicates. The levels were expressed as percentages of the mean in control pTRV plants which were defined as 100%. Means denoted by different letters are significantly different (P < 0.05) according to Tukey's test.

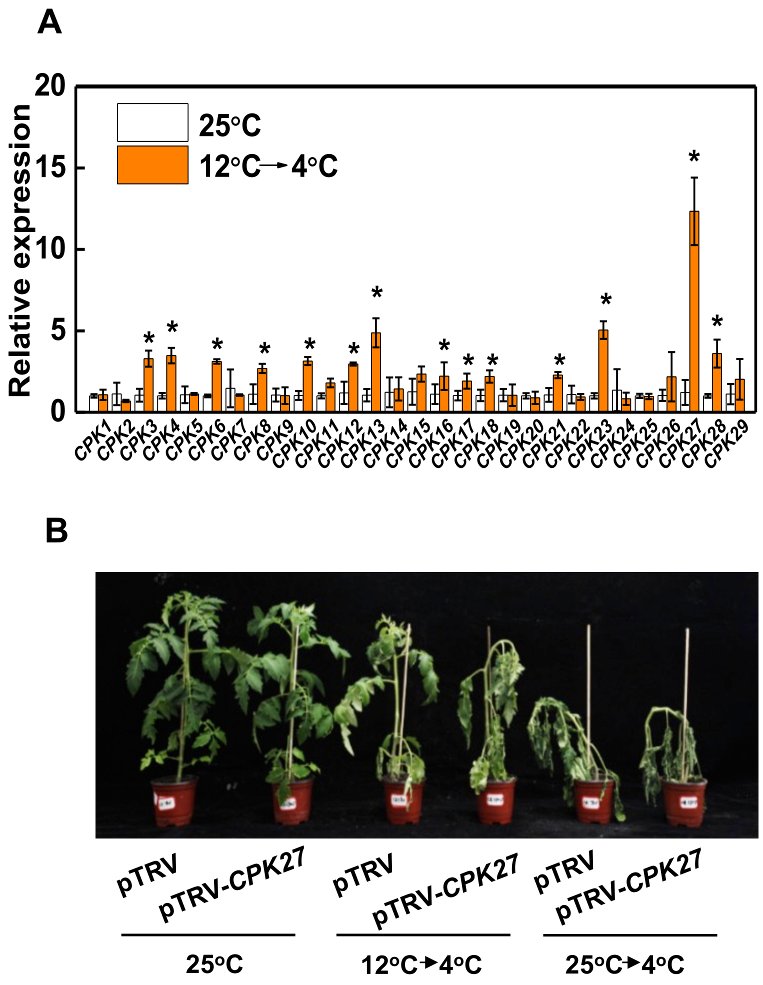

Fig. S2. Effects of cold acclimation on (A) transcripts of the tomato *CPK* family genes and (B) plant phenotype after cold stress in tomato plants. At the five-leaf stage, tomato plants were either cold acclimated (12 °C for 3 d) or kept at normal temperature (25 °C) before the imposition of cold stress (4 °C for 5 d). Transcripts of *CPK* genes were analyzed at 12 h, while plant phenotype was determined at 5 d after commencement of the cold stress treatment. Data are the means (±SD) of 4 biological replicates. *Differences from control values were significant at $P<0.05$.

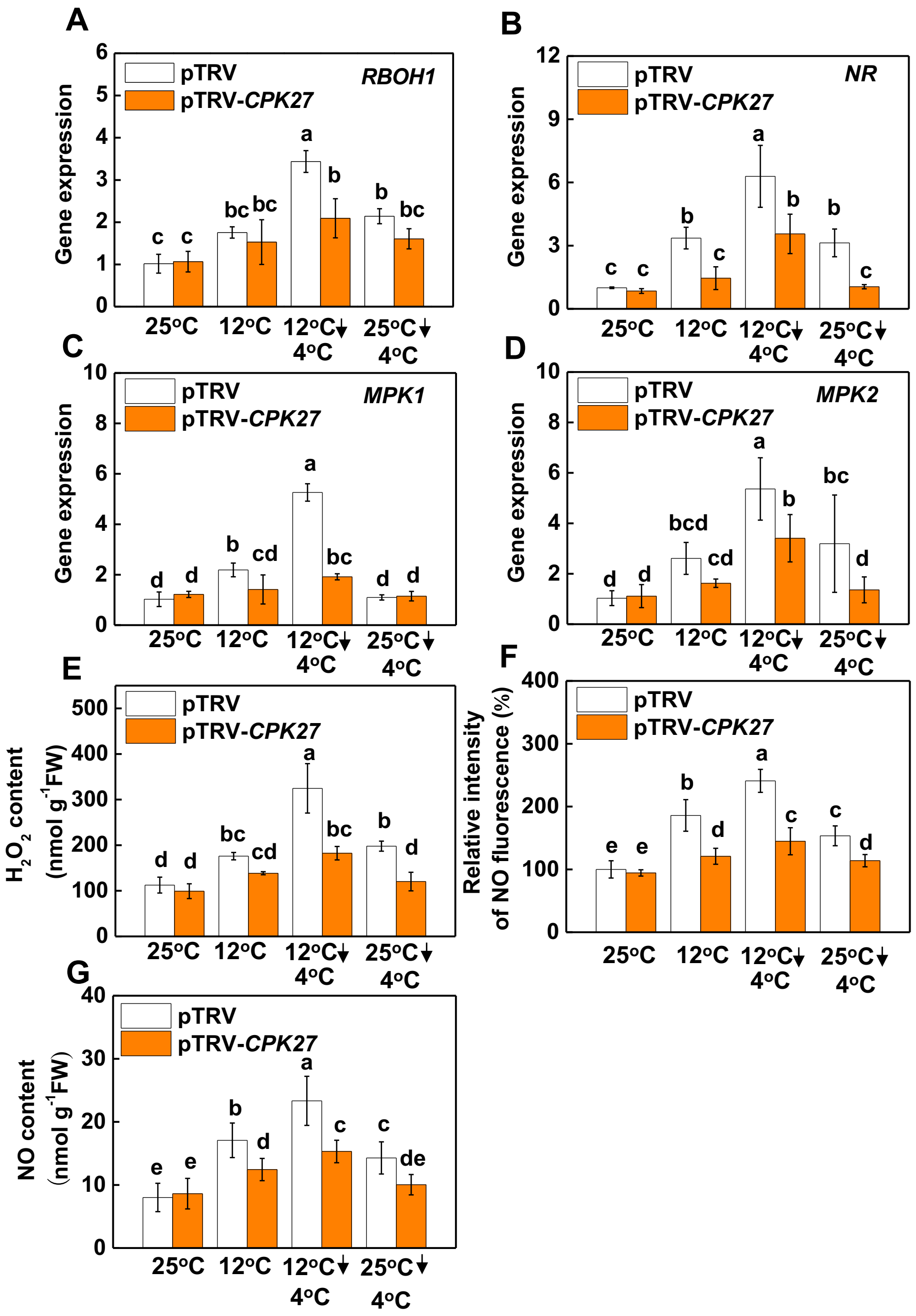

Fig. S3. Effects of *CPK27* silencing on the transcript levels of (A) *RBOH1*, (B) *NR*, (C) *MPK1*, and (D) *MPK2* and accumulation levels of (E) H_2O_2 and (F and G) NO in leaves of control (25 °C), cold-acclimated and non-acclimated tomato plants. At the five-leaf stage, tomato plants were either cold acclimated (12 °C for 3 d) or kept at normal temperature (25 °C) before the imposition of cold stress (4 °C for 5 d). H_2O_2 was extracted from leaf tissue and detected by a chemical method. NO accumulation in Fig. S3F was visualized using a NO-specific fluorescent probe, 4-amino-5-methylamino-2',7'-difluorofluorescein diacetate (DAF-2DA), and signal intensities were quantified by color histogram analysis. Meanwhile, the endogenous NO level in Fig. S3G was monitored by colorimetric assay with Griess reagent. All parameters in this figure were assayed at 12 h after commencement of the cold stress treatment. Data are the means (±SD) of 4 biological replicates. Different letters indicate significant differences (P<0.05) according to Tukey's test.

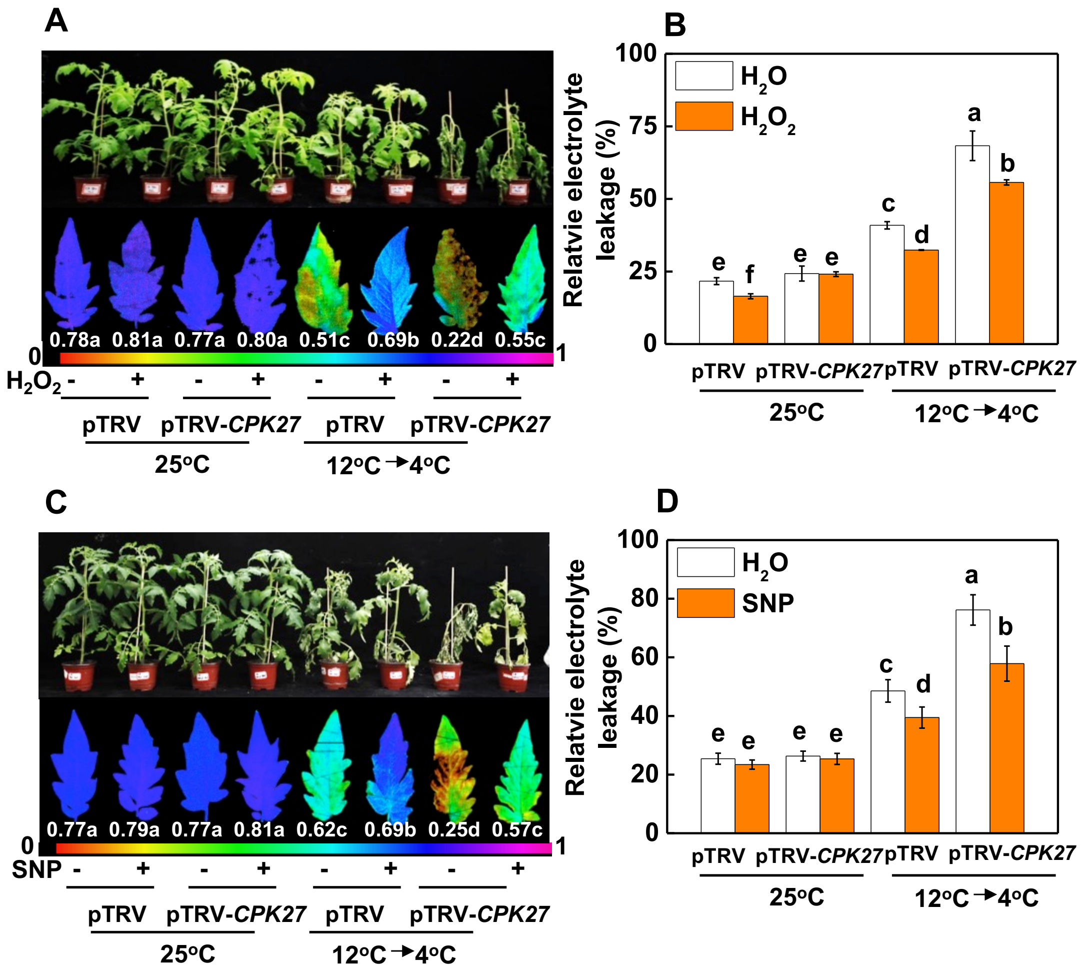

Fig. S4. Exogenous H_2O_2 or SNP partly rescues the chilling sensitive phenotype due to *CPK27* silencing. (A and C) Phenotype (upper) and the maximum quantum yield of photosystem II (*Fv/Fm,* lower) in *CPK27*-silenced plants with spraying H_2O_2 (A) and SNP(C), respectively. (B and D) REL in *CPK27*-silenced plants with spraying H_2O_2 (B) and SNP (D), respectively. The false color code depicted at the bottom of the image ranges from 0 (black) to 1.0 (purple), representing the level of leaf damage. Plant phenotype, REL and *Fv/Fm* were determined at 5 d after commencement of the cold stress treatment. Data are the means (±SD) of 4 biological replicates, except for *Fv/Fm*, which represents the mean of 15 leaves from independent plants. Different letters indicate significant differences (*P*<0.05) according to Tukey's test.

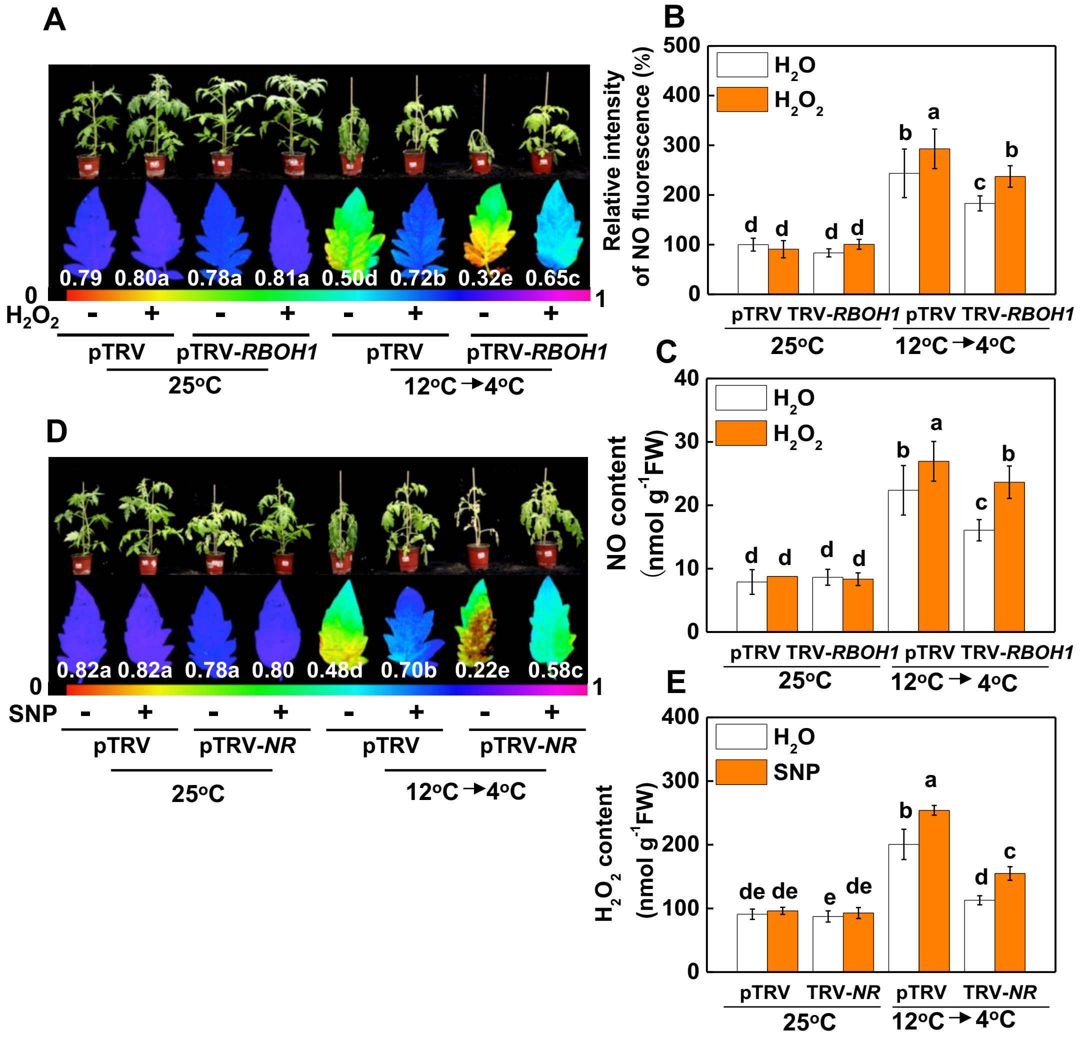

Fig. S5. Effects of *RBOH1* or *NR* silencing and exogenous H_2O_2 or SNP on cold acclimation-induced cold tolerance and NO or H_2O_2 accumulation in control (25 °C) and cold-acclimated tomato plants. (A and D) Phenotype (upper) and the maximum quantum yield of photosystem II (*Fv/Fm,* lower) in (A) *RBOH1*- and (D) *NR*-silenced plants. (B) Relative intensity of NO fluorescence in *RBOH1*-silenced plants. (D) NO level measured by with Griess reagent. (E) H_2O_2 accumulation in *NR*-silenced plants. At the five-leaf stage, tomato plants were either cold acclimated (12 °C for 3 d) or kept at normal temperature (25 °C) before the imposition of cold stress (4 °C for 5 d). H_2O_2 at 10 mM and SNP at 500 µM were applied 12 h before the cold acclimation treatment. The false color code depicted at the bottom of the image ranges from 0 (black) to 1.0 (purple), representing the level of leaf damage. H_2O_2 and NO accumulation levels were estimated as described in Fig. S3. Plant phenotype and *Fv/Fm* were determined at 5 d, while NO and H_2O_2 accumulation were analyzed at 12 h after commencement of the cold stress treatment. Data are the means (±SD) of 4 biological replicates, except for *Fv/Fm*, which represents the mean of 15 leaves from independent plants. Different letters indicate significant differences (P<0.05) according to Tukey's test.

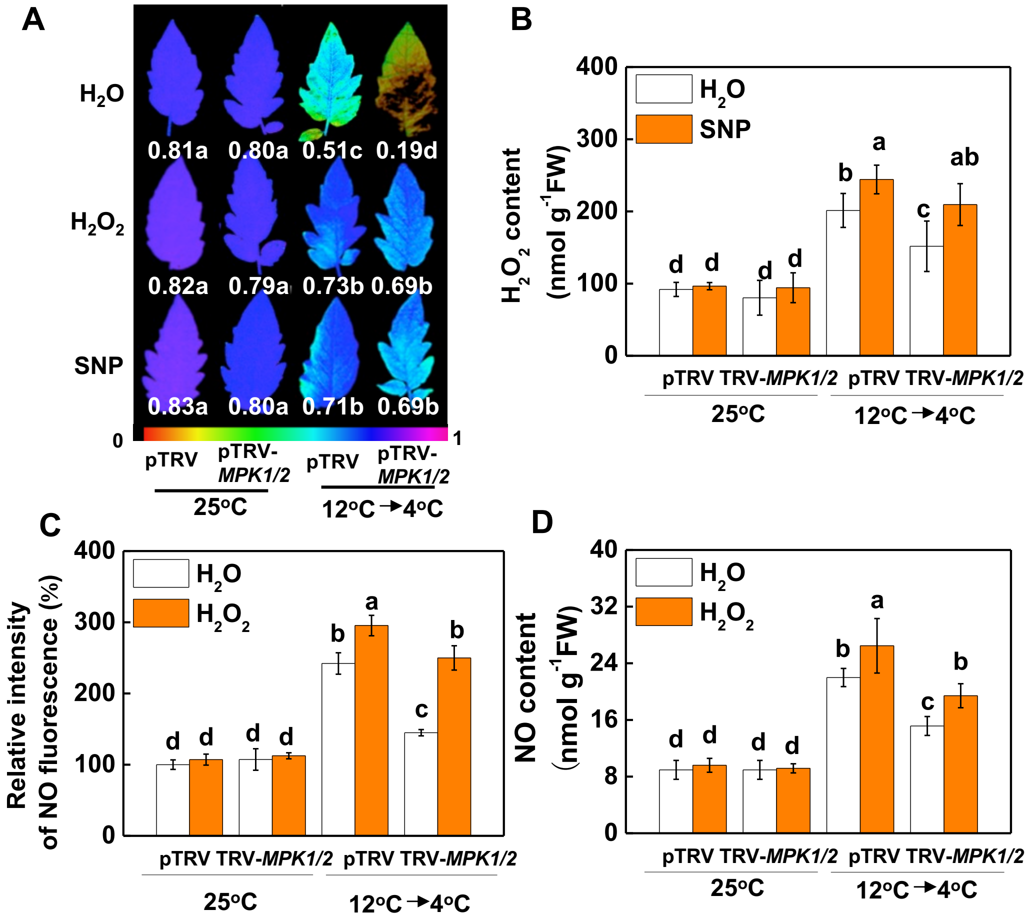

Fig. S6. Effects of *MPK1/2* co-silencing and exogenous SNP or H_2O_2 on (A) *Fv/Fm*, (B) accumulation of H_2O_2 or (C and D) NO in control (25 °C) and cold-acclimated tomato plants. At the five-leaf stage, tomato plants were either cold acclimated (12 °C for 3 d) or kept at normal temperature (25 °C) before the imposition of cold stress (4 °C for 5 d). The false color code depicted at the bottom of the image ranges from 0 (black) to 1.0 (purple), representing the level of leaf damage. H_2O_2 and NO accumulation levels were estimated as described in Fig. S3. *Fv/Fm* was determined at 5 d, while the accumulation of NO and H_2O_2 were analyzed at 12 h after commencement of the cold stress treatment. Data are the means (±SD) of 4 biological replicates, except for *Fv/Fm*, which represents the mean of 15 leaves from independent plants. Different letters indicate significant differences (P<0.05) according to Tukey's test.

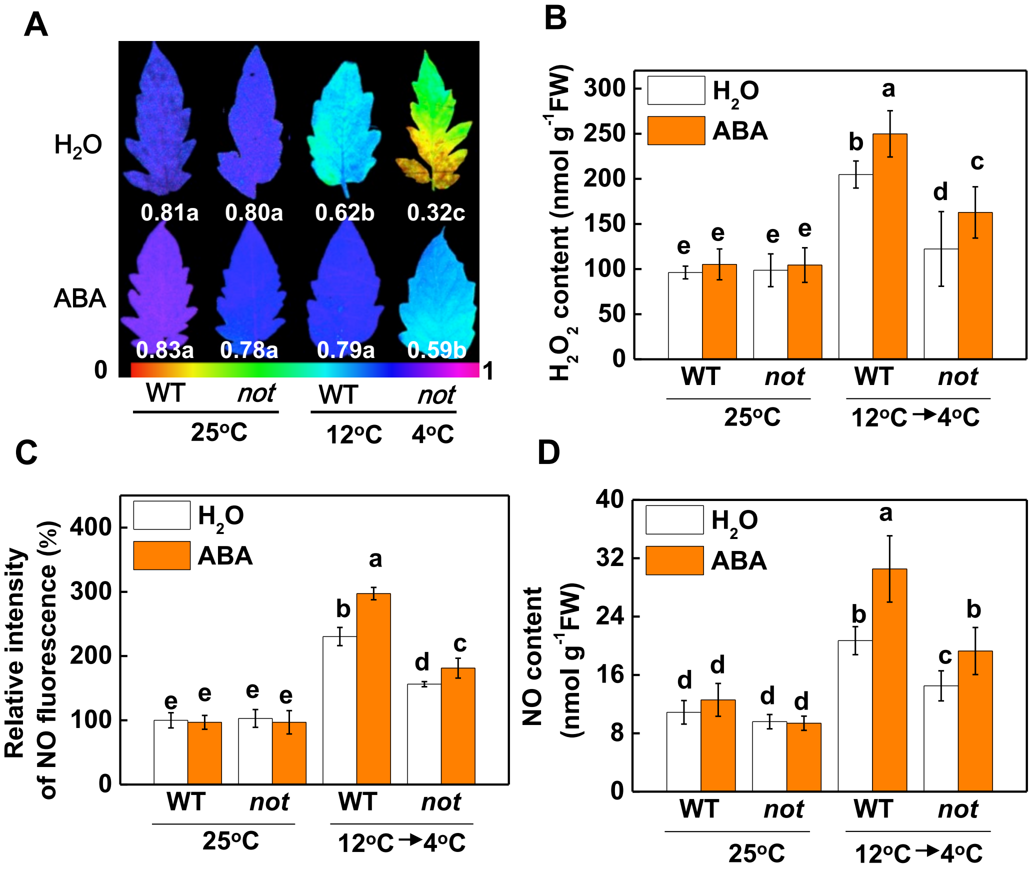

Fig. S7. ABA and cold acclimation-induced changes in (A) *Fv/Fm* and accumulation of (B) H_2O_2 and (C and D) NO in the wild type (WT) and the ABA-deficient mutant *not*. ABA at 50 µM was applied 12 h before the cold acclimation treatment. At the five-leaf stage, tomato plants were either cold acclimated (12 °C for 3 d) or kept at normal temperature (25 °C) before the imposition of cold stress (4 °C for 5 d). The false color code depicted at the bottom of the image ranges from 0 (black) to 1.0 (purple), representing the level of leaf damage. H_2O_2 and NO accumulation levels were estimated as described in Fig. S3. *Fv/Fm* was determined at 5 d, while NO and H_2O_2 accumulation levels were analyzed at 12 h after commencement of the cold stress treatment. Data are the means (±SD) of 4 biological replicates, except for *Fv/Fm*, which represents the mean of 15 leaves from independent plants. Different letters indicate significant differences (P<0.05) according to Tukey's test.

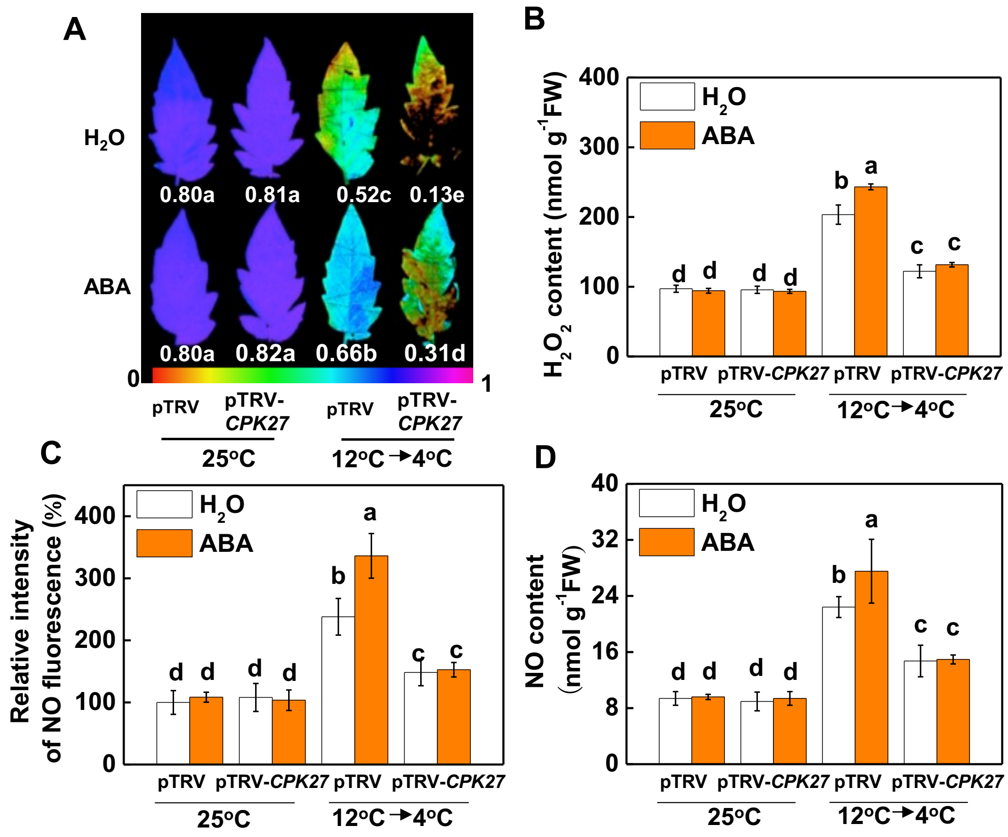

Fig. S8. ABA and cold acclimation-induced changes in (A) *Fv/Fm* and (B) accumulation of H_2O_2 and (C and D) NO in pTRV control plants and *CPK27*-silenced plants. ABA at 50 µM was applied 12 h before the cold acclimation treatment. At the five-leaf stage, tomato plants were either cold acclimated (12 °C for 3 d) or kept at normal temperature (25 °C) before the imposition of cold stress (4 °C for 5 d). The false color code depicted at the bottom of the image ranges from 0 (black) to 1.0 (purple), representing the level of leaf damage. H_2O_2 and NO accumulation levels were estimated as described in Fig. S3. *Fv/Fm* was determined at 5 d, while NO and H_2O_2 accumulation levels were analyzed at 12 h after commencement of the cold stress treatment. Data are the means (±SD) of 4 biological replicates, except for *Fv/Fm*, which represents the mean of 15 leaves from independent plants. Different letters indicate significant differences (P<0.05) according to Tukey's test.
